# Septin-Associated Protein Kinases in the Yeast *Saccharomyces cerevisiae*

**DOI:** 10.3389/fcell.2016.00119

**Published:** 2016-11-01

**Authors:** Adam M. Perez, Gregory C. Finnigan, Françoise M. Roelants, Jeremy Thorner

**Affiliations:** Division of Biochemistry, Biophysics and Structural Biology, Department of Molecular and Cell Biology, University of California, BerkeleyBerkeley, CA, USA

**Keywords:** cell cycle, cell signaling, cytoskeletal element, morphology, protein phosphorylation

## Abstract

Septins are a family of eukaryotic GTP-binding proteins that associate into linear rods, which, in turn, polymerize end-on-end into filaments, and further assemble into other, more elaborate super-structures at discrete subcellular locations. Hence, septin-based ensembles are considered elements of the cytoskeleton. One function of these structures that has been well-documented in studies conducted in budding yeast *Saccharomyces cerevisiae* is to serve as a scaffold that recruits regulatory proteins, which dictate the spatial and temporal control of certain aspects of the cell division cycle. In particular, septin-associated protein kinases couple cell cycle progression with cellular morphogenesis. Thus, septin-containing structures serve as signaling platforms that integrate a multitude of signals and coordinate key downstream networks required for cell cycle passage. This review summarizes what we currently understand about how the action of septin-associated protein kinases and their substrates control information flow to drive the cell cycle into and out of mitosis, to regulate bud growth, and especially to direct timely and efficient execution of cytokinesis and cell abscission. Thus, septin structures represent a regulatory node at the intersection of many signaling pathways. In addition, and importantly, the activities of certain septin-associated protein kinases also regulate the state of organization of the septins themselves, creating a complex feedback loop.

## Introduction

Septins are a conserved family of GTPases (Pan et al., 2007; Peterson and Petty, 2010; Nishihama et al., 2011) that serve multiple biological functions in diverse cell types (Weirich et al., 2008; Hall and Russell, 2012; Hernandez-Rodriguez and Momany, 2012; Mostowy and Cossart, 2012; Fung et al., 2014). All eukaryotes examined to date (except higher plants) encode multiple septin genes, ranging from just two in the nematode *Caenorhabditis elegans* (John et al., 2007), to five in *Drosophila melangaster* (O'Neill and Clark, 2016), to seven in *Saccharomyces cerevisiae* (Garcia et al., 2016), to 13 in humans (Peterson and Petty, 2010; Hall and Russell, 2012; Fung et al., 2014).

**Figure 1:**
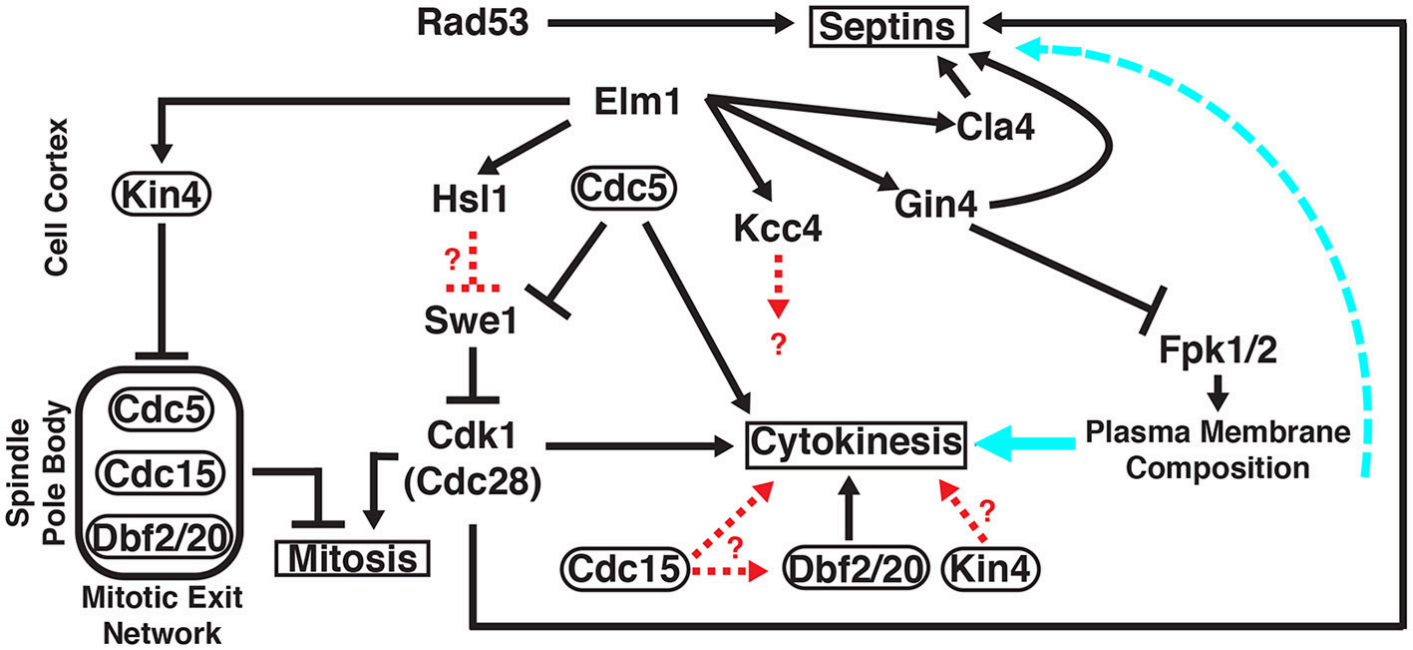
**Roles of multiple protein kinases in septin-mediated signaling networks in ***S.cerevisiae*****. Unless otherwise indicated, all of the gene products shown (Cdc5, Cdc15, Cdk1/Cdc28, Cla4, Dbf2, Dbf20, Elm1, Fpk1, Fpk2, Gin4, Hsl1, Kcc4, Kin4, Rad53, and Swe1) are protein kinases that co-localize with the septin collar at the bud neck at specific stages of the cell cycle, either very transiently or for a prolonged time period. Protein kinases that, in addition, localize at other sites are encircled by ovals. Signaling outputs of the indicated protein kinases at their non-septin locations are depicted on the left side of the panel; signaling events emanating from the septin collar itself are diagrammed on the right side of the panel. *Solid black arrows*, regulation by direct substrate phosphorylation; *dashed red arrows*, regulation exerted by unknown mechanisms; *solid cyan arrow*, influence of the plasma membrane lipid composition on the execution of cytokinesis; *dashed cyan arrow*, influence of the plasma membrane lipid composition on septin filament assembly and structural organization. See text for further details.

Budding yeast (*S. cerevisiae*) has served as a path-finding model eukaryote in which to explore the structure, function, and regulation of septins and septin-associated proteins. The products of the yeast septin genes assemble into linear, apolar hetero-oligomeric rods that are the fundamental building block of all septin-based structures (Bertin et al., 2008; Bertin and Nogales, 2012), as has now also been shown for other organisms. These rods can self-associate end-to-end to form filaments and can, depending in their subunit composition, also interact in other modes to form more elaborate super-structures, such as spirals, rings, braids, and gauze-like lattices (Rodal et al., 2005; Garcia et al., 2011; Oh and Bi, 2011; Bertin et al., 2012; Ong et al., 2014). Other factors nucleate the assembly of septin structures at discrete subcellular locations (Chen et al., 2011; Bi and Park, 2012) where they serve as both scaffolds (Shulewitz et al., 1999; Sakchaisri et al., 2004; Wloka et al., 2011; Bridges and Gladfelter, 2015) and diffusion barriers (Takizawa et al., 2000; Caudron and Barral, 2009) and thereby dictate, via direct and indirect interactions, the subcellular distribution of numerous other proteins (Gladfelter et al., 2001; McMurray and Thorner, 2009; Finnigan et al., 2016a). In particular, as discussed here, septin-based structures recruit, and thereby localize (and, in some cases, regulate the activity of) a multiplicity of protein kinases that integrate multiple inputs into signaling pathways and ultimately initiate ensuing biological responses (Figure 1).

Septins were first discovered in *S. cerevisiae* as cell division cycle (*cdc*) mutants whose compromised function resulted in abnormal growth patterns and the inability of cells to execute cytokinesis (Hartwell, 1971; Hartwell et al., 1974). Electron microscopy revealed a prominent set of filaments that encircle the yeast bud neck (Byers and Goetsch, 1976) and, later, antibody staining demonstrated that septins were constituents of those filaments (Haarer and Pringle, 1987; Kim et al., 1991). The advent of GFP and other genetically encoding fluorescent protein tags to follow protein dynamics in live cells in real time revealed further that septin organization undergoes dramatic changes during cell cycle progression. Septins first accumulate as a patch at the incipient site of bud emergence that rapidly resolves into a filamentous ring (Kozubowski et al., 2005; Okada et al., 2013), which then expands, concomitant with bud growth, into an hourglass-shaped tube or collar composed of at least 30–40 gyres of circumferential filaments at the bud neck (Byers and Goetsch, 1976; McMurray et al., 2011; Finnigan et al., 2015b; Patasi et al., 2015). At the onset of cytokinesis, the collar splits into two fllamentous bands of roughly equal size with a prominent gap in between (Dobbelaere et al., 2003; Dobbelaere and Barral, 2004) wherein factors needed for actomyosin contractile ring assembly and new plasma membrane (PM) and cell wall (CW) synthesis accumulate (Bi et al., 1998; Nishihama et al., 2009). After cytokinesis and cell separation, each daughter cell disassembles the half of the collar it inherited (Johnson and Blobel, 1999; Tang and Reed, 2002) before constructing a new septin-based site for the next bud to emerge. An N-terminal F-BAR domain—and C-terminal Muniscin/Mu homology domain (MHD)-containing protein Syp1 localizes prominently at the bud neck and has been implicated in the processes required for disassembly of the septin ring (Qiu et al., 2008). Syp1 is a highly phosphorylated protein (Albuquerque et al., 2008; Soulard et al., 2010; Swaney et al., 2013). In this regard, it is interesting that eight of the phospho-sites detected in Syp1 fit the -T/S-P- consensus for the protein kinase Cdk1/Cdc28 that is the major cell cycle driver (Verma et al., 1997; Mok et al., 2010) and five of them fit the consensus sequence (-R-x-x-S/T-) determined for the bud neck-localized protein kinase Gin4 (Mok et al., 2010; Roelants et al., 2015). However, Syp1 also localizes prominently to cortical puncta and functions as an endocytic adaptor that is involved in cargo selection and negative regulation of Las17 (yeast WASp)-Arp2/3 complex activity (Boettner et al., 2009; Reider et al., 2009) during the early stages of endocytic patch formation (Stimpson et al., 2009). Although eukaryotic proteins with such apparently disparate functions certainly exist, it is nonetheless a little hard to reconcile mechanistically these two rather distinct roles attributed to Syp1.

In *S. cerevisiae*, five (*CDC3, CDC10, CDC11, CDC12*, and *SHS1*) of its seven septin genes are expressed in mitotically-dividing haploid and diploid cells (Versele et al., 2004; Versele and Thorner, 2004, 2005), whereas the remaining two septin genes (*SPR3* and *SPR28*) are expressed only in diploid cells undergoing meiosis and sporulation (Garcia et al., 2016). The mitotic septins assemble into two types of hetero-octameric rods that differ only in their terminal subunit: Cdc11-Cdc12-Cdc3-Cdc10-Cdc10-Cdc3-Cdc12-Cdc11 (Bertin et al., 2008) and Shs1-Cdc12-Cdc3-Cdc10-Cdc10-Cdc3-Cdc12-Shs1 (Garcia et al., 2011). *In vitro* the former can associate end-to-end into paired filaments in low-salt solution and into sheets of paired filaments on the surface of a lipid monolayer containing phosphatidylinositol-4,5-*bis*phosphate (Bertin et al., 2010). Mutagenesis studies have shown that the residues needed for these characteristic *in vitro* behaviors are also essential for viability *in vivo* (Bertin et al., 2008; McMurray et al., 2011; Finnigan et al., 2015b). The Shs1-containing hetero-octamers associate laterally in a staggered fashion, rather than end-on-end, forming curved bundles, rings, and bird's nest-like structures *in vitro* (Garcia et al., 2011). Although cells lacking Shs1 are viable, the septin structures formed in its absence are aberrant (Garcia et al., 2011), most likely because, as observed *in vitro* (Booth et al., 2015), Cdc11-capped rods and Shs1-capped rods are able to form heterotypic end-on-end junctions, and likely also do so *in vivo* (Finnigan et al., 2015a,b). The dynamic interplay between these two types of hetero-octamers may facilitate the massive reorganizations of septin architecture that occur over the course of the cell cycle (Vrabioiu and Mitchison, 2006; Ong et al., 2014).

Diverse protein kinases are associated with septin structures at various points throughout progression through the cell division cycle. However, it is still not completely clear how many of these enzymes contribute directly to installing post-translational modifications on septins and/or septin-associated proteins that drive the observed dynamic changes in septin structure during cell cycle progression and how many of these enzymes are recruited to septin structures as “readers” of the status of septin assembly to phosphorylate other substrates and thereby drive subsequent downstream events. Here, we highlight key regulatory pathways that use the septin cytoskeleton as a signaling platform to direct other orchestrated events required for successful passage through the cell cycle.

## A morphogenesis checkpoint

**Figure 2:**
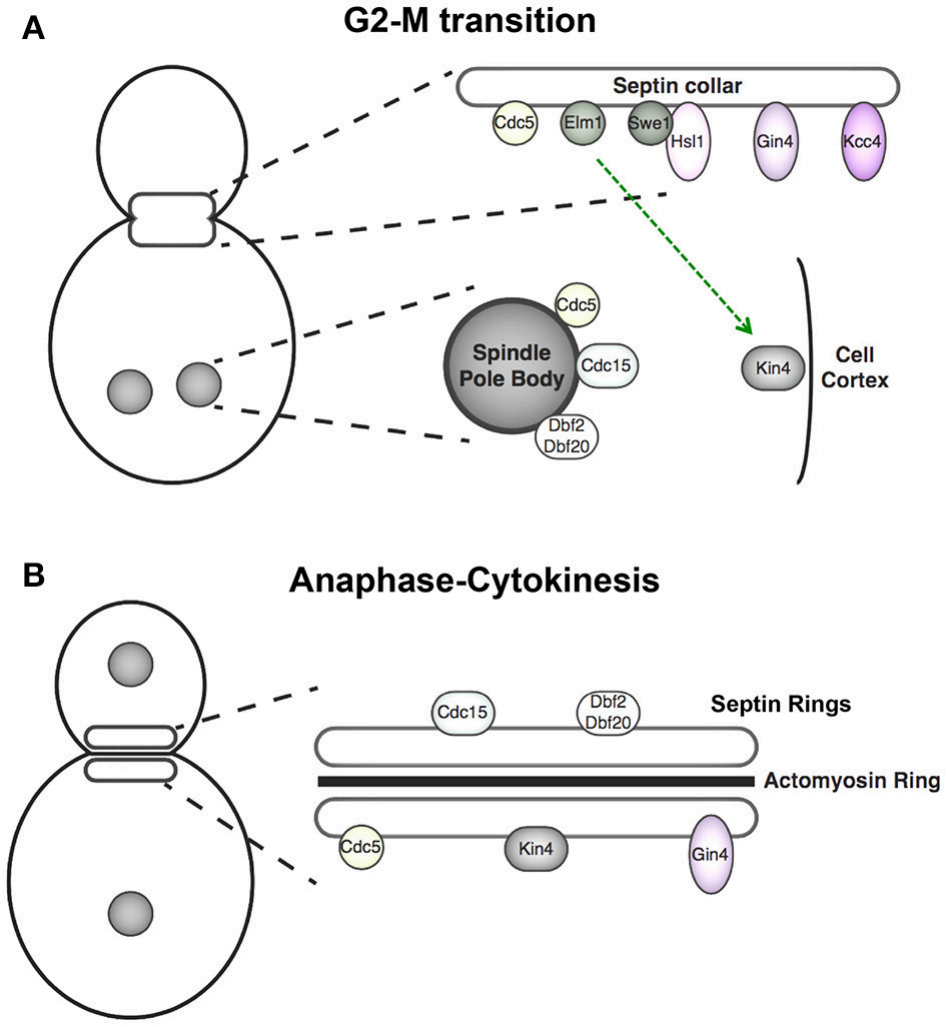
**Cell cycle-dependent localization of septin-associated protein kinases. (A)** Components of the septin-monitoring morphogenesis checkpoint localize to the septin collar at G2 where they promote Swe1 degradation, and thus full activation of cyclin B-bound Cdk1 and M phase entry. At anaphase of mitosis, the APC protein-ubiquitin ligase terminates this pathway by degrading its pivotal component, the protein kinase Hsl1. At this point in the cell cycle, the protein kinases of the MEN are located at the spindle pole bodies (SPBs) and Kin4, the protein kinase central to the spindle position checkpoint, is localized to the mother cell cortex. Kin4 and the MEN components act in concert to delay mitotic exit until one spindle pole body has been properly segregated into the daughter bud. **(B)** Upon execution of anaphase, spindle elongation, SPB segregation, and initiation of mitotic exit, the MEN components relocalize to the septin rings, where these protein kinases phosphorylate targets that help promote cytokinesis. Gin4 remains septin collar-associated from G1 through cytokinesis.

Eukaryotes have evolved quality control mechanisms, collectively dubbed checkpoints (Hartwell and Weinert, 1989; Paulovich et al., 1997; Ibrahim, 2015), by which to ensure that the events required for successful cell division are only executed at the proper time and in the proper order. Virtually all recognized checkpoint mechanisms involve regulation by reversible protein phosphorylation mediated by protein kinases and phosphoprotein phosphatases (Domingo-Sananes et al., 2011; Rhind and Russell, 2012). In yeast, a checkpoint delays the decision to pass from G2 to M phase if there is some incompleteness (or abnormality) in cell morphogenesis that needs time to be finished (or repaired). It was initially thought that this control was exerted in response to defects in actin cytoskeleton assembly and/or function (McMillan et al., 1998). However, it was demonstrated shortly thereafter that it is the state of septin collar assembly that is monitored by this checkpoint (Shulewitz et al., 1999), a conclusion that was amply confirmed subsequently (Lew, 2003; Howell and Lew, 2012). In essence, when the septin collar is properly formed, it recruits and thereby serves as a congregation point for information exchange among the regulatory factors required to release the cell from cell cycle blockade (Figure 2A, *upper*). This arrangement provides a feedback circuit by which the state of septin organization is temporally and spatially integrated with other processes necessary for cell cycle progression. Because chromosome segregation and cytokinesis cannot proceed productively if the bud neck is occluded, this checkpoint delays initiation of the G2-M transition until the septin collar has been erected properly.

In brief, the cyclin B (Clb2)-bound form of protein kinase Cdk1 (Cdc28), which is the primary driver of mitosis, is held in check by inhibitory phosphorylation on Tyr19 in its P-loop, a modification installed by protein kinase Swe1 (the *S. cerevisiae* ortholog of mammalian Wee1) (Booher et al., 1993). This phosphorylation is reversed, in part, by the action of phosphoprotein phosphatase Mih1 (yeast ortholog of mammalian Cdc25) (Russell et al., 1989). However, to lift Swe1-mediated inhibition of Cdk1 completely, Swe1 is also degraded in a highly regulated manner, as follows. In conjunction with the joint actions of the protein-arginine N-methyltransferase Hsl7 (Cid et al., 2001; Sayegh and Clarke, 2008) and the protein kinase Hsl1 (Ma et al., 1996; Barral et al., 1999; Shulewitz et al., 1999) (closest mammalian orthologs are the AMPK-related protein kinase family members, BRSK1, and BRSK2), Swe1 is exported from the nucleus (Keaton et al., 2008), captured at the bud neck, and marked there for timely cyclosome/anaphase-promoting complex (APC) protein-ubiquitin ligase-dependent ubiquitinylation (Simpson-Lavy and Brandeis, 2010; Lianga et al., 2013) and proteasome-mediated destruction via phosphorylation by protein kinase Cla4 (yeast ortholog of mammalian PAK1) and especially by protein kinase Cdc5 (yeast ortholog of mammalian Plk1) (Sakchaisri et al., 2004). Compared to Hsl1, two other septin-associated protein kinases, Gin4 and Kcc4 (which are apparent paralogs, but less related to Hsl1) have a supporting, but less significant role, in these events (Ma et al., 1996; Barral et al., 1999; Kusch et al., 2002). It should be appreciated that because Clb2-Cdc28 phosphorylation of Swe1 creates phosphosites that are docking motifs for binding the Polo boxes of Cdc5, thus priming Swe1 for Cdc5-mediated phosphorylation (Asano et al., 2005; Harvey et al., 2005), the Hsl7- and Hsl1-dependent down-regulation of the nuclear pool of Swe1 partially alleviates Clb2-Cdc28 inhibition. This initially modest increase in Clb2-Cdc28 activity then is able to initiate a self-reinforcing burst of autocatalytic activation of Cdk1 by targeting more and more of the population of Swe1 molecules for even more efficient Cdc5-mediated phosphorylation, thereby unleashing more and more Clb2-Cdk1 activity. Thus, once initiated, these processes lead to rapid hyper-phosphorylation and nearly complete destruction of Swe1 (Shulewitz et al., 1999; Sreenivasan and Kellogg, 1999).

Most importantly, Hsl1 is only active in aiding the targeting of Swe1 for destruction when Hsl1 is associated with a correctly assembled septin collar at the bud neck (McMillan et al., 1999, 2002; Shulewitz et al., 1999; Finnigan et al., 2016b). Disruption of the septin collar using mutant septin alleles results in failure to recruit Hsl1 and Hsl7 to the bud neck, thereby stabilizing Swe1, which is then able to impose a pronounced cell cycle delay that is manifest by the formation of markedly elongated buds (Shulewitz et al., 1999; Longtine et al., 2000), a phenotype that is not observed if cells lack Swe1 (Barral et al., 1999; McMillan et al., 1999; McMurray et al., 2011).

Directly fusing Swe1 to a septin subunit does not fully bypass the requirement for Hsl1 and Hsl7 in Swe1 degradation (King et al., 2012), indicating that simple tethering of Swe1 at the septin collar for subsequent phosphorylation by Cla4 and Cdc5 is not the sole function of the Hsl1 and Hsl7 enzymes. Binding of Hsl1 to septins occurs via uniquely evolved septin-binding sequences (residues 611–950) downstream of its N-terminal kinase domain, which, to operate efficiently, must act in concert with a C-terminal phosphatidylserine-binding element (KA-1 domain) (Finnigan et al., 2016a,b). Thus, Hsl1 recruitment to the bud neck likely serves as a dual “sensor,” reporting that both septin collar assembly and the plasma membrane lipid composition have achieved the proper state to initiate the destruction of Swe1 and thereby license passage from G2 to M phase. Hsl1 catalytic activity is dispensable for its recruitment to the septins (Finnigan et al., 2016b), but may be required, directly or indirectly, in recruiting Hsl7 to the bud neck (Kang et al., 2016). Hsl7 is a substrate of Hsl1 (Cid et al., 2001), and Hsl1 is also extensively autophosphorylated (Barral et al., 1999), but the functional consequences of these modifications remain to be determined.

Hsl1 (and the checkpoint) may have additional roles besides sensing the state of septin organization and plasma membrane composition. A recent study has suggested that some of these same components, in conjunction with an additional bud neck-localized protein kinase, Elm1, have a role in monitoring bud size/shape prior to licensing entry into mitosis (Kang et al., 2016). Elm1 is able to phosphorylate the activation loop in Kcc4 and Gin4 *in vitro* (Asano et al., 2006), and presumably serves as their upstream activating kinase *in vivo* (Koehler and Myers, 1997; Bouquin et al., 2000). In contrast, under the same conditions, Elm1 does not directly phosphorylate Hsl1 *in vitro* (Asano et al., 2006), but there are claims that Elm1-mediated phosphorylation is required for full Hsl1 activity *in vivo* (Szkotnicki et al., 2008). It has been suggested that Elm1 interacts with Hsl7, thereby impeding engagement of Hsl7 with Hsl1; in this way, Swe1 recruitment to the bud neck and its degradation are delayed to allow time for sufficient bud growth (Kang et al., 2016). It has been reported that localization of septins, Hsl7 and Elm1 all depend upon local membrane curvature (Bridges et al., 2016; Kang et al., 2016); it is possible, therefore, that these factors serve as sensors of cell geometry, thereby integrating this input into the timing of the decision of when to allow passage through G2-M. Although Gin4 and Kcc4 have been implicated in septin collar assembly (Longtine et al., 1998) and in the Swe1 degradation pathway (Barral et al., 1999), more recent evidence indicates that these enzymes likely exert their effects more indirectly by modulating the plasma membrane lipid distribution (Roelants et al., 2015) (see further below). Indeed, like Hsl1, Gin4 and Kcc4 also possess phosphatidylserine-binding C-terminal KA-1 domains that are required for their function *in vivo* (Moravcevic et al., 2010).

## Mitotic exit

In addition to the role of the septin collar in integrating signals that determine when to initiate entry into mitosis, growing evidence implicates septin structures at the bud neck in coordinating certain aspects of the signaling network that regulates exit from mitosis. Mitotic exit is facilitated, in large part, by a protein kinase signaling cascade, dubbed the Mitotic Exit Network (MEN) (Bardin and Amon, 2001; McCollum and Gould, 2001), that gauges successful spindle elongation by monitoring the location and status of the spindle pole bodies (SPBs) (Smeets and Segal, 2002; Hotz and Barral, 2014; Falk et al., 2016). Once anaphase has been achieved, signals emanating from the SPB in the daughter cell initiates a pathway that serves to reverse the actions of Cdk1 by unleashing the phosphoprotein phosphatase Cdc14 (Mocciaro and Schiebel, 2010; Bremmer et al., 2012). Components of the MEN regulatory circuitry in yeast have features that suggest that it may be the antecedant of the Hippo tumor-suppressor pathway in animal cells (Avruch et al., 2012; Harvey and Hariharan, 2012; Rock et al., 2013).

The core components of MEN (Figure 2A, lower) are the small Ras-related GTPase Tem1, which, in its GTP-bound state, binds to and activates the protein kinase Cdc15, which, in turn, is the upstream activator of two paralogous protein kinases Dbf2 and Dbf20 that, to be activated by Cdc15 and functional, need to associate with an essential regulatory subunit Mob1. The primary role of activated Dbf2/Dbf20-Mob1 complexes is to phosphorylate Cdc14, which both releases it from its inhibitory anchor in the nucleolus (Azzam et al., 2004) and prevents its nuclear import (Mohl et al., 2009), allowing its activity to spread throughout the cell to inactivate Clb-bound Cdk1 and reverse the Cdk1-dependent phosphorylation of many Cdk1 substrates (Visintin and Amon, 2000; Meitinger et al., 2012).

Whether Tem1 is in its inactive GDP-bound state or in its activated GTP-bound state is controlled by events at the SPB, completely independent of the septin collar. In brief, when no SPB has been segregated into the daughter bud, the Tem1 GTPase-activating protein (GAP) complex, Bfa1-Bub2, is activated by the protein kinase Kin4 located at the cortex of the mother cell and, hence, Tem1 remains in its inactive state (D'Aquino et al., 2005; Pereira and Schiebel, 2005). This regulatory scheme is referred to as the spindle positioning/orientation checkpoint (SPOC) (Caydasi and Pereira, 2012; Ibrahim, 2015). However, there is a clear connection to events at the septin collar. First, it was observed that septin-defective mutants exhibited aberrations in MEN signaling outputs (Castillon et al., 2003). Second, Kin4 (and its paralog Frk1) are, like Gin4, Kcc4, and Hsl1, members of the branch of yeast AMPK-like protein kinases (Rubenstein and Schmidt, 2007); and, indeed, it has been shown that, as for Gin4 and Kcc4, Elm1 is the upstream protein kinase responsible for phosphorylation of T209 in the activation loop of Kin4 and, further, that this modification is essential for Kin4 catalytic activity (Caydasi et al., 2010; Moore et al., 2010). Moreover, localization of Elm1 to the bud neck is critical for its phosphorylation and activation of Kin4 (Caydasi et al., 2010; Moore et al., 2010).

The action of phosphoprotein phosphatase PP2A bound to one of its two, yeast, B-type regulatory subunits, Rts1, was also implicated as a positive factor required for maintaining Kin4 at the mother cell cortex and at mother-cell SPBs (Chan and Amon, 2009). However, it was previously shown that Rts1-bound PP2A is required for maintenance of septin ring organization during cytokinesis (Dobbelaere et al., 2003). Thus, the role of Rts1-PP2A is likely indirect and simply to preserve the structure of the septin collar and, thus, the localization of active Elm1 there, supporting maintenance of adequate levels of activated Kin4 in the mother cell.

The daughter cell-specific protein Lte1, despite its sequence resemblance to other guanine nucleotide exchange factors (GEFs), does not appear to function as the GEF for Tem1. Rather, it appears to promote formation of GTP-bound Tem1 by binding to and inactivating Kin4 and excluding the kinase from the daughter SPB, thereby preventing activation of the Bfa1-Bub2 GAP (Bertazzi et al., 2011; Falk et al., 2011).

All of the MEN components, including the protein kinases Cdc5 (Sakchaisri et al., 2004), Cdc15 (Lee et al., 2001), and Dbf2/Dbf20 (Frenz et al., 2000), localize first to the SPB and then to the bud neck (Figure 2). At the SPB, Cdc5 functions in MEN by acting in concert with Tem1 to promote SPB recruitment and activation of Cdc15 (Rock et al., 2013). Cdc5 function depends on its cellular location, as revealed by experiments in which Cdc5 recruitment was artificially directed primarily to the SPB or primarily to the bud neck; at the SPB, Cdc5 was necessary for efficient mitotic exit and, at the bud neck, Cdc5 clearly promoted Swe1 degradation (Sakchaisri et al., 2004). The two C-terminal polo boxes of Cdc5 are required for stable association of this protein kinase at each of these two subcellular locations (Song et al., 2000; Lowery et al., 2005), but the protein target (s) at each location that carry the phospho-epitopes to which the polo boxes bind have not been well defined. Given the role that members of the polo family of protein kinases, including Cdc5, play in driving multiple cell cycle events subsequent to initial substrate phosphorylation by cyclin B-bound Cdk1 (Barr et al., 2004; Archambault and Glover, 2009), and the role that the septin collar plays in regulating the sub-cellular distribution of Cdc5, the coordination between septin dynamics and Cdc5 localization, as well as between septin dynamics and Elm1 localization (Thomas et al., 2003), provide regulatory inputs that contribute to ensuring that Swe1 degradation (and mitotic entry) always precedes mitotic exit. Indeed, the septin collar is the passageway through which any and all components segregated between a mother and daughter cell must pass and, hence, is a cellular structure ideally situated to monitor such cell cycle events.

## Cytokinesis

In addition to its role in tethering of factors involved in controlling the spatial and temporal aspects of the cell cycle, the septin collar also has functions in regulating membrane dynamics. At the cortex of a mother cell or its bud, where the ER is in close apposition to the PM, there are periodic intimate protein-and lipid-mediated connections between the two, referred to as PM-ER junctions (Manford et al., 2012; Gatta et al., 2015). However, at the bud neck, the septin filaments tightly coat the cytosolic surface of the PM at this location (Byers and Goetsch, 1976; Bertin and Nogales, 2012), preventing sterically the formation of such ER-PM contact sites. Although extensions of the ER can be seen to pass through the bud neck, at the location of the septin collar *per se*, the ER surface appears denuded of ribosomes and, further, that this band of smooth ER acts as a barrier to diffusion of ER membrane proteins (but not ER lumenal proteins) (Luedeke et al., 2005). On the one hand, it has been reported that establishment of this specialized ER domain and its function in restricting diffusion depends on localized recruitment of the actin—and formin-associated protein Bud6 by the septin Shs1 (Luedeke et al., 2005), whereas another more recent study claims that a bridged interaction between Shs1 and the integral ER membrane protein Scs2 is responsible for erecting this ER diffusion barrier (Chao et al., 2014). The evidence in support of both claims is not compelling for several reasons. First, more recent analysis indicates that very little of the total cellular pool of Bni6 is located at the bud neck [see entry for Bud6/Aip3/Ylr319c at the Yeast Protein Localization Plus Database (YPL+) at the Univ. of Graz, Austria; http://yplp.uni-graz.at/index.php], and Scs2 is a demonstrated component of a major class of the ER-PM junctions. Second, because *shs1*Δ cells are viable and do not display severe growth and morphology defects under most conditions (Iwase et al., 2007; Finnigan et al., 2015b), this specialized, purportedly Shs1-mediated, domain in the trans-bud neck ER doesn't seem to be very important for viability or efficient cell cycle progression. Finally, affixing the ER to the septin collar at the bud neck, and thereby risking impeding the passage of chromosomes and other cellular organelles, would not seem to make much sense physiologically for efficient cell division.

In any event, to complete each cell cycle, the spindle midbody remnant must be removed, the plasma membranes resealed, and the CW septa deposited between them, to create two, separate and independent yeast cells. In *S. cerevisiae*, cytokinesis and cell separation proceed via the concerted action of actomyosin ring (AMR) formation and contraction (Bi et al., 1998) with concomitant synthesis of the chitinaceous primary septum (Nishihama et al., 2009). To permit these events to happen following anaphase, the septin collar is split into two gasket-like bands via a mechanism that is still completely unknown (McMurray and Thorner, 2009; Bi and Park, 2012; Wloka and Bi, 2012; Juanes and Piatti, 2016). However, after septin collar assembly and release from the morphogenesis checkpoint, the elevation of Clb2-Cdc28 activity commences the process of promoting AMR formation early in M phase and, once localized to the septin collar, Cdc5 generates a local pool of activated (GTP-bound) Rho1 at the bud neck via phosphorylation and activation of one of the primary Rho1 GEFs (Tus1) (Yoshida et al., 2006). Activated Rho1, in turn, is known to stimulate formin-dependent assembly of the cytokinetic AMR and type II myosin contractility (Yoshida et al., 2006; Ramkumar and Baum, 2016). In addition, activated Rho1 also binds directly to and stimulates protein kinase Pkc1 (Kamada et al., 1996), which controls the transcription of genes needed for CW synthesis (Jung and Levin, 1999) and other events needed for cell cycle completion (Darieva et al., 2012), as well as two enzymes required for CW production (the 1,3-β-D-glucan synthase Fks1 and its paralog Gsc2/Fks2) (Mazur and Baginsky, 1996). In this way, the septins provide a platform by which the cell cycle machinery is linked to both the cytoskeletal machinery and the CW synthesis machinery that each contribute to the successful execution of yeast cytokinesis. Difficult to reconcile with these findings (and the evidence discussed earlier for its role in septin ring *disassembly*), however, is the claim that F-BAR and MHD protein Syp1 is phosphorylated in a Rho1- and Pkc1-dependent manner to promote septin collar *assembly* (Merlini et al., 2015).

The “split” septin collar appears to have two main roles. For certain proteins needed for AMR assembly, e.g., Bni5 (Lee et al., 2002), the septins continue to serve their scaffold function by mediating direct physical association with these factors, thereby anchoring and concentrating them at the bud neck (Patasi et al., 2015; Finnigan et al., 2015a, 2016a). For other proteins, e.g., Sec3 involved in localized deposition of secretory vesicles (Dobbelaere and Barral, 2004) and Chs2 involved in septum construction (Foltman et al., 2016), the two septin bands of the split collar seems to act like corrals and barriers to diffusion, thereby physically trapping these factors between them and thus confining them at this location indirectly (Dobbelaere and Barral, 2004; McMurray et al., 2011; Finnigan et al., 2016a).

In addition to Cdc5, the other two protein kinases of the MEN cascade, Cdc15 and Dbf2/Dbf20-Mob1 complexes, are recruited to the split septin rings during late anaphase, shortly after Cdk1 inactivation (Frenz et al., 2000; Song et al., 2000; Xu et al., 2000; Luca et al., 2001; Figure 2B). At this location, these enzymes recruit and phosphorylate many factors directly involved in the coordination of AMR contraction and primary septum formation. In particular, at the bud neck, Dbf2-Mob1 phosphorylates another F-BAR protein, Hof1 (Meitinger et al., 2011), which also contains a C-terminal SH3 domain important for its function (Oh et al., 2013). Hof1 associates with septin structures throughout G1-S phase and while the AMR is assembled between the rings of the split septin collar primarily via direct interaction with Cdc10 (and Cdc12) (Vallen et al., 2000; Oh et al., 2013; Finnigan et al., 2016a). Association of Hof1 with the septins is mediated by a coiled-coil element in Hof1, and phosphorylation at a single Ser within this region by bud neck-localized Dbf2-Mob1 (an event that requires priming by prior phosphorylation of Hof1 by Cdk1 and Cdc5) promotes disassociation of Hof1 from the septin rings (Meitinger et al., 2013). This displacement then allows Hof1 to associate with the AMR and initiate contraction, but the mechanism by which it does so has not been elucidated yet.

In addition to its binding to the AMR, Hof1 also forms a complex with Inn1 and Cyk3 at the septin ring in late anaphase (Sanchez-Diaz et al., 2008; Nishihama et al., 2009). Recruitment of these three components to the bud neck requires MEN signaling activity (Meitinger et al., 2010), and thus presumably their phosphorylation, and all are required for efficient cytokinesis because they contribute to coordinating AMR contraction with primary septum formation (Meitinger et al., 2012; Wloka and Bi, 2012; Juanes and Piatti, 2016). As mentioned above, formation of the primary septum requires the localized activity of chitin synthase Chs2 between the bands of the split septin collar. After Chs2 has been sequestered at this position, Dbf2-Mob1 translocates to the split rings and directly phosphorylates and activates Chs2 (Oh et al., 2012). The dynamic relocation of the protein kinases of the MEN cascade to the split septin collar provides an elegant solution to help ensure that cell division only occurs after successful chromosome segregation. However, the mechanisms that promote recruitment of these kinases to the septins are unknown. Moreover, the SPOC protein kinase Kin4 also localizes to the septin rings late in anaphase, yet its function at the bud neck is not understood.

Finally, cytokinesis cannot be completed unless and until two intact and separable PMs have been generated. In this regard, there is evidence that two types of septin-associated protein kinases spatially and temporally modulate the PM lipid bilayer assymmetry at the bud neck. At sites of polarized growth, the protein kinase Fpk1 (and its paralog Fpk2) phosphorylate and stimulate two PM-localized flippases (Dnf1 and Dnf2), which translocate phosphatidylethanolamine (PtdEth) and phosphatidylserine from the outer to the inner leaflet of the PM (Nakano et al., 2008; Roelants et al., 2010). However, at the bud neck, septin-anchored protein kinase Gin4 phosphorylates and inhibits Fpk1 (and Fpk2) function (Roelants et al., 2015). Thus, flipping of aminoglycerophospholipids is prevented in a highly localized manner. Preventing flipping of aminoglycerophospholipids in this way contributes to enhancing the efficiency of cell division because alterations that compromise flippase function suppress the inviabiity phenotype of a variety of mutations (*hof1, inn1, cyk3*, and *myo1*, the type II myosin of the AMR) known to cause defects in cytokinesis (Roelants et al., 2015). Moreover, Gin4 is released from the septin collar just before it splits and the AMR contracts, whereas the bulk of Fpk1 remains (Roelants et al., 2015), presumably fine-tuning the timing of transbilayer lipid flipping necessary for the completion of PM scission and cell separation. The mechanisms responsible for the cell cycle-coupled ejection of Gin4 and the recruitment of Fpk1 are not known, although Gin4 is phosphorylated and activated in a cell cycle-dependent manner at the G2-M transition, apparently by Clb2-Cdc28 (Altman and Kellogg, 1997) and, conversely, the septins are required for the mitosis-specific activation of Gin4 (Carroll et al., 1998). In any event, by the spatio-temporal control that Gin4 exerts on Fpk1 activity, it is clear that the septins at the bud neck are critical for protein kinase-mediated regulation of localized PM remodeling.

## Regulation of septin organization

The actions of several septin-associated protein kinases also seem to regulate septin organization. The Cdc42-stimulated protein kinase Cla4 (yeast ortholog of mammalian PAKs) directly phosphorylates septins Cdc3 and Cdc10 both *in vitro* and *in vivo*, and Cla4 is necessary for both the formation of the septin collar and the regulation of septin dynamics at specific points in the cell cycle (Dobbelaere et al., 2003; Schmidt et al., 2003; Versele and Thorner, 2004).

Septin subunit Shs1 is the most extensively phosphorylated septin and is a substrate of Gin4 (Mortensen et al., 2002), as well as of the G1 cyclin-dependent protein kinases Cdc28/Cdk1 and Pho85 (yeast ortholog of mammalian Cdk5) (Egelhofer et al., 2008). The latter marks are removed at the end of mitosis via bud neck recruitment of Rts1-bound PP2A, an event thought to aid in initiating splitting of the septin collar (Dobbelaere et al., 2003), and which is coincident with Gin4 phosphorylation of Shs1 on a distinct subset of residues (Mortensen et al., 2002; Asano et al., 2006). These data suggest that cell cycle-dependent phosphorylation states of Shs1 are key in regulating septin dynamics. However, arguing against this hypothesis, strains expressing Shs1 alleles with most of the residues phosphorylated by Cdks mutated to either to Ala or phosphomimetic Asp displayed no discernible septin-defective phenotype (Finnigan et al., 2015b). Furthermore, most of these phosphorylation sites are within a poorly conserved segment of Shs1, and deletion of this entire region does not lead to any loss of Shs1 function *in vivo* (Finnigan et al., 2015b). Perhaps phosphorylation of Shs1 by Cdks and Gin4 is redundant with additional mechanisms that regulate septin assembly, but the precise consequence of these phosphorylation events on septin structure and/or function remains unclear. In marked contrast, Shs1 is phosphorylated at a single site by the protein kinase Rad53 in response to DNA damage and replication stress (Smolka et al., 2006, 2007) and a phosphomimetic mutation of this single residue displays a prominent growth defect (Finnigan et al., 2015b), suggesting that this direct phosphorylation might represent a checkpoint whereby cell cycle progression can be delayed (via perturbation of the septin collar) to permit time for repair of the DNA damage.

The purported role that Gin4 has in proper septin collar assembly at the bud neck has very low penetrance (Longtine et al., 1998) and appears to represent only a kinetic delay (McMurray and Thorner, 2009). Given the close apposition of septin structures with the PM in the cell (Byers and Goetsch, 1976; Bertin et al., 2012) and the effect of lipids on the state of septin assembly *in vitro* (Bertin et al., 2010; Bridges et al., 2014), the phenotype exhibited by *gin4*Δ mutants might be explained by the lack of proper control of Fpk1 function and the ensuing effects on local PM lipid composition. A consequence of Gin4-mediated inhibition of flippase function (via inhibition of the flippase-activating protein kinase Fpk1) is a pronounced reduction in the inner leaflet concentration of PtdEth in the PM (Roelants et al., 2015). A low inner leaflet PtdEth level leads to activation of Cdc42 by suppressing the function of Cdc42-specific GAPs (Saito et al., 2007; Das et al., 2012), and activated Cdc42 plays a significant role in localized tethering of the factors needed for initial recruitment of septins to the site of incipient bud emergence (Iwase et al., 2006; Okada et al., 2013).

## Outlook and prospectus

Depending on their assembly state, septin-based structures provide dynamic platforms from which the action of a significant number of protein kinases can be directed both spatially and temporally. Moreover, as observed for other protein kinase-scaffold interactions (Ferrell, 2000; Good et al., 2011; Langeberg and Scott, 2015), signaling emanating from septin-associated kinases can be channeled to particular co-localized targets conferring specificity and can be insulated from improper substrates to ensure fidelity. Moreover, where necessary, co-recruitment of multiple protein kinases permits signal propagation in the appropriate sequence and enables cross-talk to elicit coincident and combinatorial outputs. Moreover, these septin structures serve as sensors that transmit upstream cues, such as cell cycle timing and membrane curvature, to their associated kinases. Certain of these kinases also regulate septin structure and organization, establishing an extremely complex feedback system which is yet to be fully understood. As highlighted through the course of the above discussion, there are still many mechanistic aspects of the control of septin-associated protein kinases that remain to be delineated. Hence, this area of cell biology and biochemistry remains a fertile area for exploring the role of cellular structures in regulating signaling enzymes, and vice versa.

## Author contributions

AP, GF, and FR prepared the draft and JT edited and submitted the final version of the manuscript.

### Conflict of interest statement

The authors declare that the research was conducted in the absence of any commercial or financial relationships that could be construed as a potential conflict of interest.
